# Current strategies against COVID-19

**DOI:** 10.1186/s13020-020-00353-7

**Published:** 2020-07-09

**Authors:** Shahid Hussain, Ya-Jia Xie, Dan Li, Shaukat Iqbal Malik, Jin-cai Hou, Elaine Lai-Han Leung, Xing-Xing Fan

**Affiliations:** 1 State Key Laboratory of Quality Research in Chinese Medicine, Macau Institute for Applied Research in Medicine and Health, Macau University of Science and Technology, Macau, SAR China; 2 Department of Bioinformatics and Biosciences, Capital University of Science and Technology, Islamabad, Pakistan; 3 Beijing Wante’er Biological Pharmaceutical Co., Ltd., No. 32 Yard, East 2nd Road, Yanqi Economic Development Zone, Huairou District, Beijing, China

**Keywords:** COVID-19, Therapeutic strategies, Traditional Chinese medicines, Immune-therapy

## Abstract

Coronavirus disease (COVID-19) caused by severe acute respiratory syndrome coronavirus 2 (SARS-CoV-2) recently was declared a pandemic by world health organization (WHO) Due to sudden outbreaks, currently, no completely effective vaccine or drug is clinically approved. Several therapeutic strategies can be envisaged to prevent further mortality and morbidity. Based on the past contribution of traditional Chinese medicines (TCM) and immune-based therapies as a treatment option in crucial pathogen outbreaks, we aimed to summarize potential therapeutic strategies that could be helpful to stop further spread of SARS-CoV-2 by effecting its structural components or modulation of immune responses. Several TCM with or without modification could be effective against the structural protein, enzymes, and nucleic acid should be tested from available libraries or to identify their immune-stimulatory activities to enhance several antiviral biological agents for effective elimination of SARS-CoV-2 from the host. TCM is not only effective in the direct inhibition of virus attachment and internalization in a cell but can also prevent their replication and can also help to boost up host immune response. Immune-modulatory effects of TCMs may lead to new medications and can guide us for the scientific validity of drug development. Besides, we also summarized the effective therapies in clinical for controlling inflammation. This review will be not only helpful for the current situation of COVID-19, but can also play a major role in such epidemics in the future.

## Background

Emerging and a sudden pathogen outbreak has always been a challenge in public health and the economic sectors worldwide. Coronaviruses belong to *coronoviridae* family and order nidovirales, which has none segmented positive-sense RNA [1] and are widely found in birds, humans, and other mammals. Six species are most common in human, in which NL63, HKU1, OC43, and 229E causes common cold symptoms while two other strain including Middle East respiratory syndrome coronavirus (MERS-CoV) reported in the Middle East in 2012 [2] and severe acute respiratory syndrome coronavirus (SARS-CoV) observed in 2003 in Guangdong Province, China, are zoonotic and are highly pathogenic resulting in fatal diseases in humans [3]. In December 2019, China faced another destructing SARS-CoV-2 outbreak leading to major health issues. The first cases with unknown etiology were reported at the end of December 2019 from Wuhan Hubei, China [4]. The causative agent was announced by Chinese authority in the first week of January 2020 as a novel coronavirus 2019. A first viral genome sequence was issued on 10 January 2020 [5], followed by submission of the other four genomic sequences confirmed the indicating association of the virus with the severe acute respiratory syndrome (SARS) [6]. In the light of available evidence, SARS-CoV-2 is considered to be transmitted from wild animals with the most possible from bats directly or maybe via other intermediate animals, confirmation of clear source (s) will help to identify the pattern of transmission [7]. *Rhinolophus* bat is mostly suspected which is found in south china and also in other Asian countries in abundance. This evidence is supported by studies found 500 CoVs identification in bats in China [8]. Bats are exceptional food available in a few Chinese restaurants and local markets. After the primary transmission, the rapid rate of infection spread due to direct transmission from humans to humans in families, health care centers and public contact areas [9]. Signs and symptoms of COVID-19 patients include dry cough, fatigue, fever, dyspnea runny nose, and in some cases nasal congestion but fever is still considered to be a typical symptom [10]. In such sudden outbreaks, diagnostic challenges always remain a problem before proceeding to treatment. Initially, biochemical tests from respiratory biological samples (bronchial aspirates, sputum, bronchoalveolar lavage fluid, nasal and pharyngeal swabs) are helpful to differentiate and specifically identify particular viruses including influenza, parainfluenza virus, MERS-CoV, SARS-CoV, adenovirus, avian influenza [11]. Molecular techniques in comparison with Biochemical tests are found the most accurate and successful ways in pathogen identification. To confirm SARS-CoV-2 through real-time RT-PCR is importantly recommend and used. Several other advanced technologies like metagenomic next-generation sequencing (mNGS) is also implemented to rapid diagnosis of COVID-19 [12]. These approaches not only help medical experts to study the entire infectome i.e. Bacteria, RNA virus, or DNA virus within the infected organism but are also important routes toward disease prevention and treatment [13]. At the time of writing this review article, no effective vaccine or drugs are available for the treatment of COVID-19, worldwide scientific communities are working die-hard to contribute in outbreak control, some of them under trail dugs and immune modulators are previously published shown in Table 1 [14].

**Table 1:** Therapeutic options for COVID-19 [59]

Diseases	Drug targets	Antiviral agents	Status	Target pathogens	RefS.
SARS-CoV-2; MERS-CoV	Spike glycoprotein	Nafamostat	Approved	Anticoagulant therapy in Asian countries	[99, 100]
SARS-CoV-2; SARS-CoV; MERS-CoV	Interferon response	Recombinant interferons	Approved	Metastatic renal cell carcinoma (IFN-α2a)	[101–103]
			Approved	Melanoma (IFN-α2b)	
			Approved	Multiple sclerosis (IFN-β1a, 1b), chronic granulomatous disease (IFN-γ)	
SARS-CoV-2; SARS-CoV; MERS-CoV	Endosomal acidification	Chloroquine	Approved	Malaria and certain amoeba infections	[99, 104–106]
			Open-label trial	SARS-CoV-2	
Broad-spectrum(e.g. coronaviruses; SARS-CoV-2)	Interferon response	Nitazoxanide	Approved	Diarrhea	[99, 107]
Broad-spectrum (HCoV-229E)	Interferon response	Cyclophilin inhibitors (Compound 30)	Preclinical	–	[108]
Influenza; SARS- CoV-2	RdRp	Favipiravir	Approved	Influenza	[99, 109]
			Randomized trial	SARS-CoV-2	
SARS-CoV; SARS-CoV-2; RSV; HCV MERS-CoV	RdRp	Ribavirin	Approved	HCV and RSV	[99]
			Randomized trial	SARS SARS-CoV-2	
SARS-CoV-2	RdRp	Penciclovir	Approved	HSV	[99]
SARS-CoV-2; MERS-CoV; SARS-CoV		Remdesivir (GS-5734)	Phase 3	SARS-CoV-2	[99, 109, 110]
			Phase 1	Ebola	
SARS-CoV-2; MERS-CoV; SARS-CoV; HCoV-229E; HIV; HPV	3CLpro	Lopinavir	Approved	HIV	[110] [103, 111]
			Phase 3	SARS-CoV-2	

**Fig. 1:**
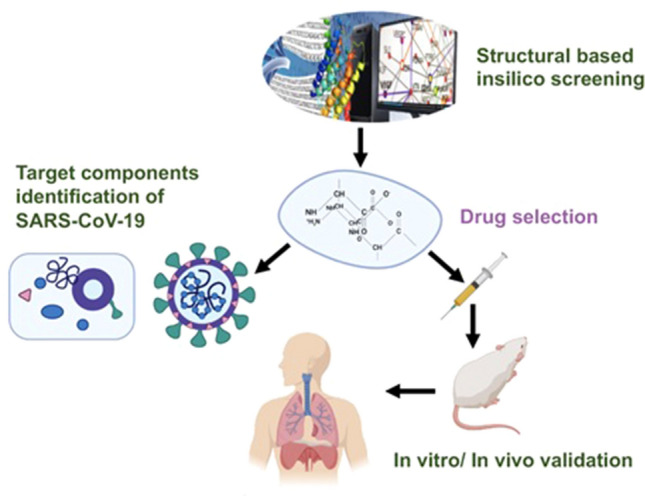
Generic representation of candidate drug targets and selection for in vitro and in vivo studies of SARS-CoV-2

To overcome the further spread of SARS-CoV-2, precautions and preventions are highly recommended i.e. to restrict traveling to affected areas, direct personnel exposure to a diseased individual, use of disinfectants in routine, and least exposure of human to human. Since no completely effective treatment is available for COVID-19, but in this regard, China has approximately two thousand old histories of using traditional Chinese medicines (TCM) against most of the viral infections including large-scale cold outbreaks being effective since Zhong-Jin Zhang (AD150- 219) [15]. So TCM could be a better hope in the recent outbreak as well as in future such epidemics, a generic drug screening and selection process are shown in Fig. 1. Few examples relevant to respiratory viral infections e.g. Yin-Qiao-San and Jing-Fang-Bai-Du-San dispelling wind and cold, Ma-Xing-Shi-Gan-Tang can remove toxic heat obstruction in the lungs so has been effective in past [16], Further investigation and research in TCM can also contribute in providing therapies for COVID-19. TCM is not only effective indirect inhibition of virus attachment and internalization in cell thus prevent their replication and further spread but also boost up host immune response [17] and can enhance T cell proliferation, antibody production, expression of pathogen-specific CD8 +  and CD4 + response, the release of IgA, IgG2 and IgG1, increase Th1 type cytokine secretion, activation of alveolar macrophages, suppress Th2/Th17-responses and keep Th1 and Th2/Th17 cells balanced thus preventing further severity [18]. From the available reports SARS-CoV-2 outbreak has infected thousands of individuals with high mortality rate due to unavailability of effective therapies, with this in mind we aimed to summarize some important therapeutic strategies to enhance host immune response with the help of traditional Chinese medicine, which are observed quite effective against viral epidemics in past before effective vaccines were developed for the complete virus elimination so the implementation could be a hopeful contribution in COVID-19 therapeutic strategies.

## Traditional Chinese Medicines (TCM) as antiviral drugs

Traditional Chinese medicine (TCM) and natural medicine have unique advantages in antiviral, which can directly affect the virus and inhibit its proliferation and can also regulate the host immune response, reinforce or inhibit the specific and non-specific immunity function, or induce and promote the secretion of IFN acting as antiviral. Because of some significant characteristics (multi-component, multi-target, system therapy), TCM plays a critical role in the course of COVID-19 treatment. But the major fact to be counted is low toxicity and high efficiency of new antiviral drugs identification from natural compounds. In the case of COVID-19, several initial problems may exist but it may provide help to clinicians in giving suitable advice after the pharmacologic effects, drug interactions, adverse drug effects, validation of study, clinical effects, and indications and limitations are resolved. Currently, the use of TCM formulas and herbs are the right choice according to the early indications matching the molecular mechanisms and pathophysiology of the disease [19].

### TCM for viral infective treatments in clinics/antiviral components in TCM

TCM is composed of many antiviral active components including alkaloids, flavonoids, glycosides, volatile oil, phenols, etc. Polyphylla saponin-I has antiviral effects on the influenza A virus [20]. Ginsenosides has a protective effect on the lethal infection of a Hemagglutinating virus of Japan (HVJ), as well as has antiviral activity on the hepatitis A virus, coxsackievirus B3, enterovirus 71, and human rhinovirus 3 [21]. Ginsenoside Rg3 attenuates hepatitis B virus DNA replication through the degradation of TRAF6/TAK1 and the inhibition of JNK/AP-1 signaling [22], and Rg3 inhibits hepatitis C virus (HCV) infection via restoring the HCV-induced dynamin-related protein 1-mediated aberrant mitochondrial dynamics [23]. Glycyrrhizic acid is effective against infection of coxsackievirus A16 and enterovirus 71 while Glycyrrhizin has an inhibition effect on the replication of the SARS-associated virus [24]. Radix Glycyrrhizae restricts infection of the human respiratory syncytial virus through preventing viral attachment, internalization, and by stimulating the secretion of IFN [25]. Flavonoids are a class of ingredients with good anti-inflammatory and antiviral activities and widely found in varieties of TCM and natural medicine. Houttuynia cordata is a traditionally used medicinal plant for the treatment of pneumonia, and flavonoids are one of the main antiviral bioactive constituents including quercetin and isoquercetin. Houttuynia cordata Thunb (HCT) blocks HSV infection through inhibition of NF-κB activation [26]. HCT water extract exhibited significant inhibitory effects on SARS-CoV and regulates immunity through stimulating the proliferation of mouse splenic lymphocytes, increases the proportion of CD4^+^ and CD8^+^ T cells, and the secretion of IL-2 and IL-10 [27]. HCT alleviates H1N1-induced acute lung injury in mice by inhibition of influenza neuraminidase activity and Toll-like receptor signaling. Baicalin, a flavonoid, inhibits the influenza virus by modulating NS1-mediated cellular innate immune responses [28]. Baicalein triazole inhibits respiratory syncytial virus (RSV) -infection and prevents pulmonary airway inflammation through the activation of the IFN signaling pathway. Moslae herba flavonoids attenuate IAV-induced barrier dysfunction by inhibiting NOX4/NF-κB/MLCK pathway [29].

### Mechanism and current antiviral TCM formula

The positive role of TCM has been emphasized in the “Diagnosis and treatment of novel Coronavirus Pneumonia (Trail Version 6)”, and some Chinese patent medicines are recommended to treat COVID-19. Xuebijing injection (XBJI) is a Chinese patent medicine that was approved for the treatment of sepsis in China [30]. XBJI shows a protective effect in patients with severe pneumonia, which mainly inhibits the activation of the TLR4-NF-κB pathway and decreases the secretion of pro-inflammatory cytokines, such as TNF-α, IL-6, IL-8, and IL-17, etc. [31]. XBJI also can improve the coagulation function to reduce inflammation and oxidative stress damage, and inhibit pulmonary fibrosis. XBJI significantly improves the primary endpoint of the pneumonia severity index in critically ill patients with severe community-acquired pneumonia [32]. Xiyanping injection (Approval ID: 2010-03, XYPI), the major bioactive ingredient is sulfonated andrographolide, has a significant effect on acute viral infection of the respiratory system, foot and mouth disease, mycoplasmal pneumonia in children [33]. Shenfu injection (SFI) reduces the levels of the pro-inflammatory cytokine of IL-6, IL-8, and TNF-α, while increases the anti-inflammatory cytokine levels of IL-4 and IL-10. SFI reinforces immunity function by regulating the expression of complements and cytokines levels [34]. A study has shown that SFI alleviates acute lung injury and improves the survival rate by suppressing the HMGB1-NF-B pathway in a rat model of endotoxin shock, thus preventing cytokine storm [35]. Xingnaojing injection (XNJI), flavonoids are its main components, improves cerebral ischemia/reperfusion injury through inhibiting the inflammatory response by the SIRT1 pathway and regulating oxidative stress [36].

## Suppression of inflammatory cytokines

The increased release of many cytokines is directly involved in the initiation and progress of inflammation caused by exogenous pathogens, including IFN-γ, IL-6, and TNF-α, etc. The excessive secretion and release of cytokines in a hyper-inflammatory state may result in a dangerous condition known as “cytokine storm”, that is associated with disease severity [37]. Clinical studies show that the levels of IL-2, TNF-α, IFN-γ, IP-10, MCP-1and other pro-inflammatory cytokines are significantly increased in severe or critical patients with COVID-19, while SARS-CoV-2 infection also increases secretion of anti-inflammatory cytokines (IL4 and IL10) [38]. The balance of pro-inflammatory and anti-inflammatory cytokines is essential for maintaining immune homeostasis. Therefore, some therapeutic interventions against these pro-inflammatory cytokines and chemokines may contribute to alleviating adverse inflammatory reactions. Concerning COVID-19 an “inappropriate and weak immune response” appears more frequently in patients with comorbidities. Thus, this could favor virus replication and enhance complications related to severe cases of the disease [39]. In patients having COVID-19, the levels of chemokines and cytokines are noted abnormal which includes vascular epithelial growth factor (VEGF), hepatocyte growth factor (HGF), TNF-α, MIP 1-α, MCP-1, IP-10, IFN-γ, GM-CSF, G-CSF, M-CSF, IL-17, IL-13, IL-12, IL-10, IL-7, IL-6, IL-1, IL-2 and IL-4 [40]. The main factor of the COVID-19 is the decrease of the immune response of the innate immune system against viruses and also the increased production of inflammatory cytokines [39].

### The role of IFNs in antiviral activities

Interferon (IFNs) induces the expression of interferon-stimulated genes (ISGs) for defense against numerous viral infections. IFNs have been classified into three major subfamilies: type I IFNs (comprising mainly IFN-α and IFN-β), type II IFNs (IFN-γ), and the recently identified type III IFNs (IFN-λ). Type I interferons(T1-IFN)have immune defense effect on many viral infections [41] and are a critical effector of the innate immune response following virus infection. IFN-α/β is a pluripotent inflammatory cytokine typically induced by viral infections. IFN-α is produced by leukocytes while IFN-β is a fibroblast product. IFN-α suppresses classical swine fever virus (CSFV) replication through Interferon stimulated gene 15 (ISG15) mediated Beclin-1 (BECN1) ISGylation and autophagy inhibition [42]. IFN-α inhibits acute IAV replication and controls the excessive immunopathology caused by IAV. A recent study showed that IFN-α and IFN-β both have protective effects during acute chikungunya virus (CHIKV) infection, but the mechanisms are different. IFN-α inhibits CHIKV replication, whereas IFN-β prevents CHIKV pathogenesis by alleviating inflammation induced by neutrophils [43]. IFN-γ, as type II interferons are released by activated T cells, natural killer cells (NK cells), and NKT cells. IFN-γ plays an important role in both innate and adaptive immunity. IFN-γ is a hallmark cytokine of type I helper T cells (Th1 cells), which have antivirus, immune regulation, and antitumor properties. Type III IFN or IFN-λ is mainly as an epithelial cytokine, which inhibits viral replication in epithelial cells and has a protective effect on mucosal sites [44]. IFN-λ exhibits biological characteristics, signal transducer, and effector functions similar to T1-IFN. IFN-λ also acts directly or indirectly on NK cells to potentiate their activation and protect against viruses. Studies have shown that IFN-λ controls multiple gut viruses and is a potent innate immune regulator of intestinal viral infections, such as rotavirus, reovirus, norovirus, enterovirus, parvovirus, and coronavirus [45]. IFN-λ restricts Influenza A virus (IAV) replication and alleviates infection‐induced morbidity, while prevents the potential inflammatory complications associated with IFNα [46]. IFN-λ also suppresses the replication of severe acute respiratory syndrome coronavirus (SARS-CoV) in both lung and gastrointestinal tracts. IFN-λ3 has anti-HBV activities and immune-modulatory effects to restrict HBV replication. Preclinical studies show that IFN-λ effectively inhibits HCV replication and has fewer side effects than IFN-α due to its more restricted receptor distribution [47]. While treating the inflammatory diseases biological agents are effective sources, but they have some limitations which includes the loss of response and cost. Also they may have large number of side effects in patients. Here an example is that many patients do not tolerate chimeric antibodies because of the hypersensitivity reactions to the agents which are non-human components. There is another issue regarding immunosuppression which results in increasing the microorganisms’ infections so in patients the latent infections may reactivate such as tuberculosis, Hepatitis B and Pnuemocystis pneumonia. As these agents should be administrated subcutaneously or intravenously so there is also an issue regarding their administration because of the reaction of the site of injection [48].

### Role of interleukin in virus infection

Interleukin is a family of cytokines with bidirectional immune-modulatory effects, mainly involved in the differentiation and activation of immune cells. IL-12 family, as inflammatory factors, includes IL-12, IL-23, IL-27, IL-35, and IL-39. IL-12 is a key immune-regulatory cytokine, which bridges nonspecific innate resistance and antigen-specific adaptive immunity [49]. IL-12 plays a crucial role in type 1 immunity, which induces many cytokines and inflammatory responses. IL-12 has a protective effect and increases the resistance to infection in herpes simplex virus type 1(HSV-1)-thermally injured mice. IL-12 could down-regulate PD-1 and increase CD8 functionality for immunity in persistent HBV infections [50]. IL-23 is a pro-inflammatory cytokine as well as IL-12, which promotes the proliferation of T cells and memory T cells and the production of IFN-*γ*. IL-23 enhances IFN-α responsiveness to promote HCV eradication. IL-23 promotes host resistance vaccinia virus infection via the IL-23/IL-17 axis. IL-23 produced by myeloid dendritic cells induces SOCS1 expression and causes T cell dysfunction during HIV infection [51]. IL-27 is secreted by antigen-presenting cells such as macrophages and dendritic cells (DCs), which has a dual role in immune regulation [52]. As an anti-inflammatory role, IL-27 could induce IL-10 producing Tr1 cells capable of inhibiting Th1 and Th17 type responses, but also as a pro-inflammatory cytokine to break down CD4^+^ Tregs or by activating Th1 differentiation [53]. IL-27 has broad anti-viral effects, such as HIV-1, HCV, herpes simplex virus type 1 (HSV-1), and HBV, but the mechanism differs. IL-27 could prevent macrophages from HIV-1 infection by down-regulating spectrin β none-erythrocyte 1 (SPTBN1). IL-27 is capable to suppress HCV replication by inducing the activation of STAT-1 and it can also control HSV-1 infection via the up-regulation of STAT-1, IL-6, and IP-10 and MIG [54]. IL-35, a newly identified member of the IL-12 family, suppresses immunity by regulatory T and B cells and plays an important role in the immune tolerance period of viral infection. IL-35 induces immune-tolerance through suppressing pro-inflammatory cytokine expression during chronic HBV infection [55]. IL-35 contributes to prevent HCV-induced liver damage via reducing inflammatory responses, while play a contradictory role in persistent HCV infection by the inhibition of antiviral immune activity. IL-35 is the high expression in peripheral blood mononuclear cells and throat swabs of patients with seasonal IAV, which inhibits IAV RNA replication and viral protein synthesis by induction of type I and III IFN [56]. Overall, IL-35 plays a critical role in inflammatory and autoimmune diseases, but the functions of IL-35 in antiviral infections are not yet well understood. IL-15 is critical for the development and function of natural killer (NK), NKT, and memory CD8^+^ T cells. IL-15 has innate antiviral activity and is an activator of NK cell-mediated antiviral defense [57]. IL-15 could enhance the killing ability of NK cells while contributing to the synthesis and secretion of IFN. Therefore, IL-15 potentially stimulates NK cells from HIV-positive donors and improves NK cells’ antiviral effects to clear HIV-1-infected cells. IL-15 efficiently enhances the survival and effector function of HIV-specific CD8^+^ T cells, which may improve their activity to control HIV [58]. IL-15 is up-regulated during primary human immunodeficiency virus (HIV) infection, which renders primary human CD4^+^ cells more susceptible to HIV. IL-15 and IFN-γ restraints HCV replication via the ERK pathway [59]. Synergistic effects of IL-15 and IFN-α significantly enhance the CD8^+^ T cell response in a state of immune hypo-responsiveness. IL-22 is a member of the IL-10 family of cytokines. It has been documented that IL-22 has a crucial role in many virus infections, including HBV, rotavirus, HIV, influenza virus, dengue virus, and HSV-2 [60]. IL-22 induces the proliferation of liver stem/progenitor cells via activating STAT3 and reduces pathology in mice and patients with chronic HBV infection. IL-22 could enhance the expression of the interferon-stimulated gene (ISG) and limit rotavirus replication by cooperating with IFN-λ [61]. After the influenza virus infection, IL-22 promotes epithelial cell regeneration, while prevents lung inflammation and secondary bacterial infection. IL-22 could reduce the secretion of pro-inflammatory cytokines and the accumulation of neutrophil to restrict dengue virus-induced hepatic damage and inflammation [62]. Studies have indicated that IL-22 has the ability to antiviral by the activation of the JAK/STAT signaling pathway [63].

## Therapeutic approaches by targeting virus structural components

**Fig. 2:**
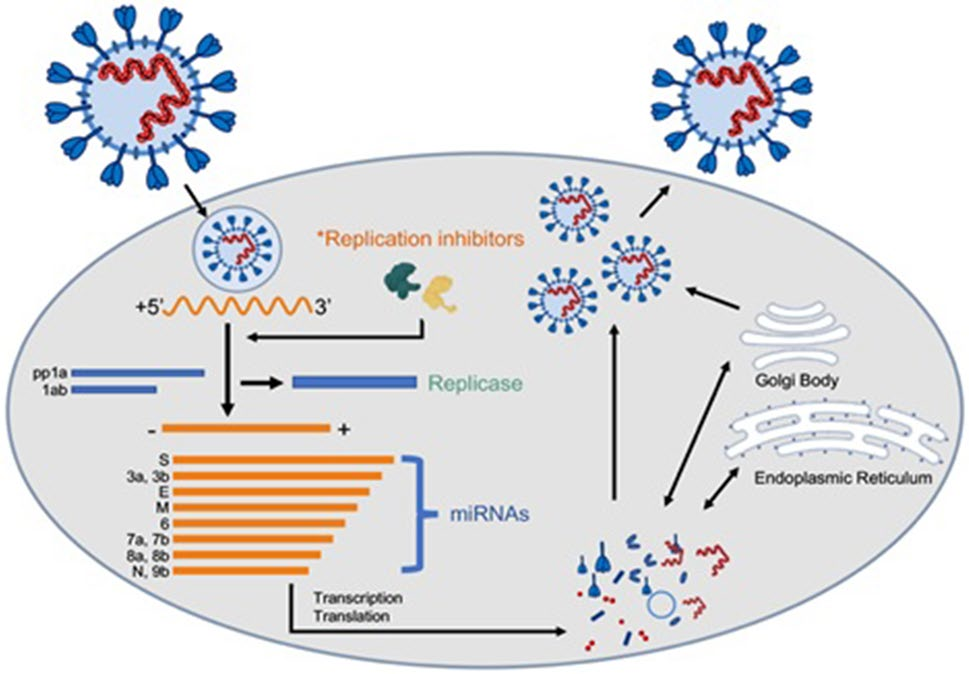
Therapeutic agents can interfere to inhibit SARS-CoV-2 life cycle by targeting the replication process

SARS-CoV-2 encodes structural and non-structural proteins including papain-like protease, RNA-dependent RNA polymerase, 3-chymotrypsin-like protease, helicase, spike glycoprotein, and some other accessory proteins. Some of these enzymes are responsible for the viral life cycle and attachment to host cells for penetration. These structural components were also identified in MERS and SARS as the target for drug development [64]. Initial analysis of structural protein and genetic sequences indicated that drug binding sites are conserved in SARS, SARS-CoV-2 and MERS, therefore repurposing of available inhibitory drugs for SARS and MERS could be effective against SARS-CoV-2 [14]. AP2-associated protein kinase 1 (AAK1) is one of the regulators in endocytosis. Janus kinase inhibitor baricitinib has the affinity to inhibit AAK1, thus could prevent COVID-19 acute respiratory disease by reducing virus entry and inflammation [65]. Host Kinases are much important in initiating antiviral signaling cascades regulating the expression of cytokines and chemokines resulting in infection prevention. Another type of kinases involves c-Jun N-terminal kinases 1 and 2 (JNK1/JNK2) which regulates initiation of pro-inflammatory responses leading to the production of interleukin 6 (IL-6), TNF-α and interferon β (IFN-β) [66]. Similarly, upon viral infection early cytokines dysregulation is an outcome of high pathogenesis, p38 MAPK is crucial in hypercytokinemia so targeting p38 MAPK could be helpful in the antiviral drug development approach [67]. G-protein coupled receptor kinase 2 (GRK2) is a potential pro-viral host protein for influenza A virus and is inhibited by paroxetine acting as virion un-coating can reduce viral load in respiratory tracts but is not helpful in lethal infection prevention [68]. In another study treatment of mice with SphK1 or SphK2 inhibitors prolonged survival by decreasing RNA synthesis of influenza A virus [69], thus could be effective in RNA synthesis inhibition in SARS-CoV-2 as presented in a predictive unidentified agents in Fig. 2. However, the safety and efficacy of these strategies mentioned above must be further investigated for clinical use.

### Antibodies based therapy

**Fig. 3:**
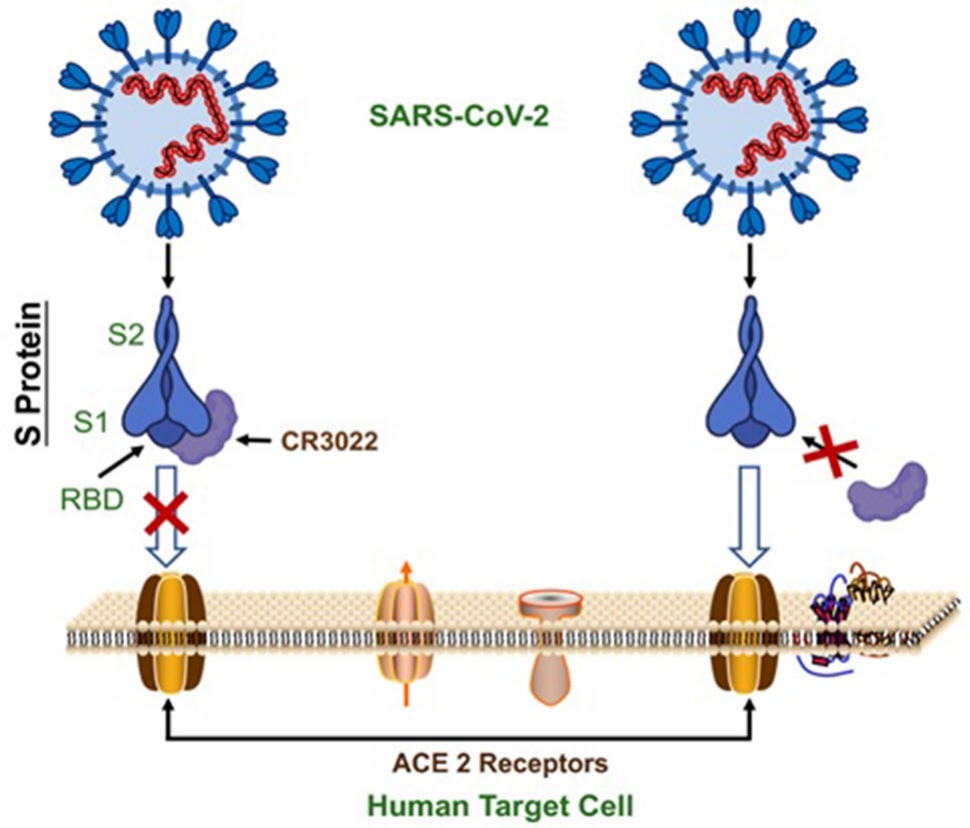
Inhibitory mechanism of antibody (CR3022) on SARS-CoV-2 attachment to ACE-2 receptor

Antibodies are the key biological molecules of the immune system, effective in minimizing viral pathogenesis because of their specificity to host protein. Monoclonal antibodies are much specific in their activities and have been tested against fatal pathogenic viruses like cytomegalovirus, Ebola, HIV-1, and influenza and approximately in the last two decades, 60 recombinant antibodies have been developed [70]. Another study has shown that RBD-43E4 and RBD-14F8 chimeric antibodies have an effective role in neutralizing MERS-CoV. Similarly, monoclonal antibodies were characterized and produced against the spike protein of MERS-CoV. cELISA was developed using one of those monoclonal antibodies. cELISA was then used to detect the specific antibodies of MERS-CoV in the sera from infected rats and rabbits which were immunized with the spike protein of MERS-CoV [71]. While the combination of two human monoclonal antibodies CR3022 and CR3014 are effective in case of SARS-CoV and also these two antibodies can extend protection [72]. When the spike protein’s conserved region binds with the human monoclonal antibodies, it is very appropriate for generating therapeutic antibodies and also for the protection against a large number of SARS-CoV variants. Similar to Influenza, HIV, and RSV, SARS-CoV-2 also has trimeric spikes protein (TSP) on the envelope. SARS-CoV-2 trimeric spikes protein tends to bind ACE2 of the host cell receptor and facilitate viral entry. By targeting TSP effective neutralizing antibodies could be effective to inhibit viral attachment to host cells. CR3022 is a SARS-CoV-specific human monoclonal antibody and its epitope does not overlap with ACE2 binding site within SARS-CoV-2 RBD and could have the potential to bind with SARS-CoV-2 RBD as illustrated in Fig. 3, therefore this alone or with the combination of other neutralizing antibodies could be a therapeutic candidate for SARS-CoV-2. MERS-CoV, SARS-CoV, and SARS-CoV-2 are all strains of coronaviruses so following the same pattern with some required modification could be helpful in antibodies development for complete neutralization of SARS-CoV-2 in human. There are some limitations based on the function of the therapeutic antibodies including the tissue accessibility, insufficient pharmacokinetics, and also the reduced interactions with the immune system. Because of these limitations there is a need of more research on these antibodies [73].

### Peptides based antiviral therapy

Antiviral peptides have a strong potential as a therapeutic drug for infection prevention. A single peptide can be verified for broad-spectrum antiviral activity. The first known example of a cationic peptide is α-defensin having antiviral activity against cytomegalovirus and herpes simplex virus, while human neutrophil peptide 1 (HNP1) with the in vitro significant effects against vesicular stomatitis virus. Due to high viral mutation probability traditional vaccines are limited in certain epidemics, in such scenario vaccination strategies based on antibody-mediated protection can stop virus infection, but high mutation rates make escape virus for host infection, Therefore classical antibody-based vaccines are proven weak in inducing T cell response. The inclusion of small peptides that could be presented by MHC molecules to T cells can produce antigen-specific responses [74]. By following this concept, a multi epitope peptide vaccine has been found effective against SARS-CoV-2 by a potential triggering of CD4^+^ and CD8^+^ T cell immune response [75]. Peptides based antiviral strategies may work in different aspects like entry blocker peptide helps in the prevention of viral fusion to host cells, virucidal peptides act upon virus envelope, and disrupt the structure and viral replication inhibitors can block further division [76]. A major significance of Peptides based therapy is that there are fewer chances of resistance development. On the other hand SARS-CoV-2 spike RBD is different from SARS-CoV spike RBD which supports the use of existing therapeutic peptides for the SARS-CoV spike RBD against SARS-CoV-2 [77]. But peptides based therapy has some limitations like low oral bioavailability and shorter half-life, Peptide hydrophilicity has poor permeability through physiological barriers and biological membranes, rapid digestion by proteolytic enzymes of the digestive system and blood plasma as well as rapid clearance from the circulation within few minutes by the liver and kidneys, Poor specific bio-distribution due to high conformational flexibility, High production costs of synthetic peptides, Therapeutic peptides and proteins also tend to undergo denaturation, aggregation, and adsorption which limit their active concentration and proper function in vivo [78].

## Other therapeutic options

Host-based antiviral therapies include enhancement of interferon responses, targeting host factors, and host signaling pathways facilitating virus replication. These approaches are significant to reduce inflammation severity and also keep immune reactions balanced to act against pathogens effectively. Both adaptive or innate immune responses can be targeted by these approaches [79]. After the complete structural and molecular composition is fully explored, several pre-existing or newly identified immune-modulatory agents with the combination of an effective antiviral may be a potential therapeutic option against COVID-19. Here we have summarized some of the important compounds related to host immune-based antiviral therapy which could be indicated helpful if used with or without modification against COVID-19 after investigation. Mesalazine and celecoxib are COX inhibitors having wide-ranging clinical applications for having analgesic, anti-inflammatory, and antipyretic characteristics, but many studies have found the application of these inhibitors (celecoxib, and mesalazine) in combination with zalamivir are more effective in reducing cytokines/chemokines levels and mortality in mice but due limited existing knowledge in present, these are not recommended in severe respiratory viral infections caused by SARS-CoV-2 [80]. On the hand Corticosteroids are steroid hormones bind with cytoplasmic corticosteroid receptor which is involved in regulating anti-inflammatory genes transcription mechanism, therefore, could be used in anti-inflammatory treatment as experienced in H1N1 influenza pandemic in 2009 resulting in treatment of 40% infected individuals having acute respiratory distress syndrome in France while 62.2% H7N9 avian influenza-infected patients were treated with corticosteroid in china during 2013 [81]. In case of SARS-CoV, MERS-CoV, and SARS-CoV-2 infections, cytokines are highly induced causing lung injury due to inflammation, in such patients corticosteroids were used frequently for viral clearance but have found to be not significantly effective in controlling mortality, therefore, more investigation is needed to confirm the harm or benefit of systematic corticosteroid based COVID-19 treatment [11]. Reactive oxygen species (ROS) also have a role in viral replication and inflammatory responses thus antioxidants having anti-inflammatory and antiviral activities could be also tested to treat cytokine storm. Antioxidants monotherapy has limited effects on cytokine storm so the combination with antiviral is still needed to be studied [82]. Increased oxidative stress because of rapid free radicals release, use of antioxidants like vitamin C can play a role in COVID-19 management [83]. A high level of Angiotensin II in SARS-CoV-2 patients is related with lung injury and viral pathogenesis, so several angiotensin receptor blockers could have potential again SARS-CoV-2 [84] Statin is an angiotensin receptor blockers (ARBs) is proposed to be an immune modulator agent for reducing inflammatory responses and is also an inhibitor for 3 hydroxy-3-methylglutaryl coenzyme A (HMG-CoA) reductase, exerting immune effector cells by ROS inhibition, and can inhibit pro-inflammatory cytokine expression in some viruses. Statin in combination with caffeine is more effective in viral replication inhibition and alleviation of lung injury and has a similar activity like ribavirin and oseltamivir [85]. Since we have no report found that shows the role of OX40 (CD134) against SARS-CoV-2. OX40 (CD134) can play a major role in T-cell-mediated immunotherapy against viral infection in the lungs through up-regulating anti-apoptotic gene expression [86]. Along with anti-pathogenic actions some antiviral and antibiotics also possess immune-modulatory properties and anti-inflammatory activities e.g. Macrolides reduced viral replication and reduced inflammatory cell number [87]. Arbidol is also an antiviral agent which might also be effective in the treatment of severe viral infection and reducing inflammation by modulating the expression of pro-inflammatory cytokines and is approved in Russia and China for their inhibitory effects on SARS-CoV-2 [88]. Interestingly in TCM, plant extracts from Jiawei-Yupingfeng-Tang can also have a big contribution against viral infection by modulating immune effects and can alleviate viral-induced lung lesions with both antiviral and immune-modulatory activity [89]. *Pseudomonas aeruginosa* injection (PA-MSHA) is a kind of biological agents for immunotherapy, which exhibits a broad immune-modulatory effect and is used for adjuvant treatment of malignant tumors in clinical. Studies have demonstrated that PA-MSHA promotes DCs maturation and enhances T-cell activation. PA-MSHA, also as an adjuvant, activates natural immunity by the Toll-like receptor (TLR) pathway and improves HIV-1 DNA vaccine immune response [90]. SARS-COV-2 infection in severe and critical stage can cause viral sepsis. In addition, PA-MSHA effectively inhibits the release of pro-inflammatory factors, reduces inflammatory response and tissue injury, and lowers the rate of infection complications in sepsis. PA-MSHA also can reverse the drug resistance [91]. Therefore, PA-MSHA may play a role in regulating immune response and inflammation in COVID-19.

## Conclusion

Because of the recent epidemics of SARS-CoV-2 all over the world, it leads to a huge loss in health and economic sectors. In past, two such epidemics of respiratory complications were observed with the other two strains of coronavirus termed as MERS-CoV and SARS-CoV. In all such outbreaks initially, symptom treatment is focused to be clinically managed in existing ill patients as well as further spread of the fatal pathogen. Globally public health authorities are mainly focused on quarantine affected individuals; restrict travelers from epicenters, disease control measures, transmission, and prevention to overcome further mortality until the development of complete effective medications for COVID-19. No doubt these are effective ways in disease management, but this is not a long term possible way to control SARS-CoV-2 further spread due to everyday interactions in public especially china with a high population rate in the world. Recently along with many other ongoing clinical trials in the field of TCM, three Chinese patent drugs, Jinhua Qinggan granules (approval number of State Food and Drug Administration of China (SFDA) Z20160001), Lianhua Qingwen granules (SFDA approval number Z20100040), and Xuebijing injection (SFDA approval number Z20040033) have been approved by the National Medical Products Administration of China and have proved inclusion for the treatment of the novel coronavirus pneumonia, which has become the world’s first batch of drugs suitable for COVID-19 [92, 93]. Similarly, another concluded that the treatment of COVID-19 patients with either arbidol (200 mg t.i.d.), nebulized IFN-α2b (5 mU b.i.d.), or a combination of arbidol plus IFN-α2b. IFN-α2b with or without arbidol can effectively decrease the duration of the virus in the upper respiratory tract and also decrease the duration of the high blood levels for the CRP and inflammatory markers IL6 [94]. In outbreaks of H5N1, Ebola, and SARS, effective neutralizing antibodies have been generated for passive immunotherapy with satisfactory results so this technique has importance in the treatment of COVID-19. A major role in the development of passive immunotherapy with plasma has been experienced with ensured hyperimmune immunoglobulins or monoclonal antibodies [95]. In the current outbreak convalescent plasma therapy in uncontrolled studies in China indeed indicated a beneficial treatment for patients on mechanical ventilation [96]. The same technique of passive immunotherapy is also been practiced in many other countries. Based on the therapeutic options mentioned in this review several TCM containing bioactive compounds can target structural components and directly inhibit attachment, penetration, and replication as well as can counteract through stimulation of T cells, antiviral cytokines, and production of antigen-specific antibodies for the elimination of SARS-CoV-2. Already some other agents like fusion peptide (EK1) [97], anti-inflammatory drugs (hormones and other molecules), RNA synthesis inhibitors (such as TDF, 3TC) and abidol [98] are effective in vitro. Therefore more clinical investigation has to confirm its efficacy and safety against SARS-CoV-2 and meanwhile some drug options could be adopted from past treatment experience of SARS and MERS. However the application of TCM in COVID-19 needs to be studied both in vivo and in vitro to identify exact anti-SARS-CoV-2 compounds, mechanism of action, antiviral efficacy, potential synergistic effects, and immune-stimulatory effects to prioritize and select a drug for a potential application in the ongoing COVID-19 outbreak.

## Data Availability

Not applicable

## Abbreviations

TCM: Traditional Chinese medicine
COVID: Coronavirus disease
WHO: World health organization
MLCK: Myosin light-chain kinase
TLR: Toll-like receptor
PA-MSHA: Pseudomonas aeruginosa-mannose-sensitive hemagglutinin
RBD: Receptor binding domain
IFN: Interferon
IL: Interleukin
CHIKV: Chikungunya virus
